# TPE-optimized XGBoost model for fine–grained prediction of tertiary vocational college students’ employment destinations

**DOI:** 10.1371/journal.pone.0359421

**Published:** 2026-09-28

**Authors:** Juntao Chen, Jinmei Zhan, Xiaodeng Zhou, Jiahua Yao, Su-Kit Tang, Xia Liu

**Affiliations:** 1 School of Financial Management, Hainan College of Economics and Business, Haikou, China; 2 Faculty of Applied Sciences, Macao Polytechnic University, Macao, China; 3 School of Information Science and Technology, Qiongtai Normal University, Haikou, China; 4 Faculty of Humanities and Social Sciences, Macao Polytechnic University, Macao, China; 5 Sanya Aviation and Tourism College, Sanya, China; Southwest Petroleum University, CHINA

## Abstract

Predicting tertiary vocational college students’ employment destinations has become an urgent need for most tertiary vocational colleges. This study proposes a machine learning framework based on Tree-structured Parzen Estimator (TPE)-Optimized eXtreme gradient boosting (XGBoost) for fine-grained prediction of tertiary vocational college students’ employment destinations. The dataset comprises 2,270 graduates (2016–2019 cohorts) from a single tertiary vocational college in China, characterized by 27 features spanning demographics, entrance scores, academic records, and extracurricular engagement. Employment destinations are categorized into three fine-grained classes: smooth employment, further study, and unemployment (class distribution: 78.8%, 17.8%, 3.4%). Recursive Feature Elimination (RFE) reduced the feature set to 22 informative variables. TPE hyperparameter optimization (100 iterations) was applied to the XGBoost classifier using an 80/20 train-test split, with stratified 5-fold cross-validation to mitigate overfitting risk. The proposed model achieves an overall accuracy of 81.72% and a balanced accuracy of 48.80% on the held-out test set, with macro-averaged precision of 0.6608, macro-averaged recall of 0.4880, macro-averaged specificity of 0.7659, and macro-averaged F1-score of 0.5263, outperforming seven baseline classifiers, including non-optimized XGBoost, logistic regression (LR), decision tree (DT), support vector machine (SVM), random forest (RF), K-nearest neighbor (KNN), and LightGBM. SHAP analysis identifies academic GPA across multiple semesters, Gaokao scores, and volunteer service hours as the most influential predictors.

## Introduction

It is widely acknowledged that the employment rate and quality of graduates are key indicators of a university’s educational performance and talent–training effectiveness. In particular, graduate destination is a critical dimension of employment quality, carrying profound implications for individuals, institutions, industry and society [1]. Recently, there has been growing interest in leveraging machine learning to predict student’s employment outcomes. Through large–scale surveys, Garcia-Penalvo et al. identify employability as a central pillar of European higher education strategy [2], while Ouyang et al [3] regarded the forecasting of graduate trajectories (employment versus further study) as a priority research domain. Zhao, He, and Feng [4] examined the mobility patterns and determinants of graduate employment against the backdrop of massification and Chen Dongrui et al [5] highlight the gap between expected and actual graduate salaries, noting vulnerability to social narratives and entrepreneurial pressure. Byeon [6] mapped occupational mobility among Korean graduates, and Ye et al [7] provided multi–institutional assessments of labour–market outcomes. Owing to these remarkable studies, this hold promise for a broad range of studying employment, e.g., predicting tertiary vocational students’ employment destinations. Many experimental efforts have been devoted toward investigating the university students’ employment. Theoretical and experimental works have been directed towards measuring and characterizing the university student’s variable. For instance, Yang [8] adopted logistic regression for variable significance screening and tested SVM, Naïve Bayes, classification and regression trees (CART), RF and XGBoost, incorporating predictors such as discipline, degree level, region, industry and gender. Wei et al. [9] inputted into SVM with gender, employment structure, student leadership experience, academic achievement and career planning indicators, using prediction accuracy and feature count as a composite fitness function. Their studies may be more reasonable if they had tertiary vocational college student considered this situation. Their results could be better convinced.

Tertiary vocational education, a cornerstone of advanced manufacturing, still lacks precise predictive tools capable of reconciling reported employment rates with realized job placements [10]. Wei et al [9] attribute the growing employment mismatch to surging graduate numbers, economic slowdown and pandemic after shocks, all of which threaten social stability and human capital formation. Yang [8] critiques conventional graduate behaviour studies for their reliance on questionnaires methods that are inherently subjective and prone to low data fidelity. Existing literature predominantly employs classical statistics or rudimentary machine learning techniques, these approaches exhibit insufficient accuracy and robustness.

When it comes to research on establishing predictive models using students’ employment history information, a number of studies have demonstrated that various machine learning methods have achieved good performance in terms of both foresight and accuracy [3,4,11–13]. Recent advances in machine learning have also explored unsupervised and deep learning approaches for educational outcome prediction. For instance, graph-based autoencoders [7] and contrastive learning frameworks [3] have demonstrated strong representation capacity on large-scale student data. More broadly, machine learning has proven effective across diverse prediction and classification domains, including climate-based disease incidence modeling [14], pharmacokinetic modeling enhanced by transform-based machine learning [15], early quality prediction in manufacturing [16], predictive and analytical solutions in complex systems [17], and optimizer-assisted kernel extreme learning machines for classification [18]. These studies collectively underscore the versatility and robustness of optimization-assisted machine learning, further motivating the TPE-optimized XGBoost framework adopted in this work. However, these approaches typically demand large datasets and substantial computational resources, limiting their direct applicability to small-scale institutional data. In contrast, this study focuses on a small-scale dataset, where an interpretable, optimization-based gradient boosting framework provides a more practical and deployable solution for individual tertiary vocational colleges. In this study, small data refers to structured, limited-volume data collected specifically for defined research or administrative purposes [19]. Unlike big data, it is static rather than real-time, sampled rather than exhaustive, and typically has high quality with few or no missing values. The key innovations of this study are: (1) fine-grained three-class employment destination prediction tailored for tertiary vocational education; (2) integration of RFE-based feature selection with TPE hyperparameter optimization in a unified pipeline; and (3) deployment of SHAP for destination-specific interpretability to support data-driven educational management.

## Literature review

The employment destinations of tertiary vocational college students are shaped by a wide range of key determinants, and existing research has leveraged basic student profiles and training process data to predict student academic performance [20]. Extant literature has established that machine learning (ML) has emerged as a powerful approach for data mining and decision support in student performance research, with broad applications in domains including early employability assessment [21], academic self-efficacy evaluation [22], and student performance prediction [7].

Early research has consistently shown that tree-based supervised machine learning algorithms are particularly well suited for educational datasets due to their transparent structure and robust interpretability [23,24]. XGBoost is a high-performance tree-based ML method that has been widely applied across diverse fields, including starting salary estimation [24], employment density analysis [25], prediction of asphalt rheological properties [26], and assessment of potential soil and groundwater contamination risks from gas stations [27]. In recent years, the application of ML technologies in educational prediction has expanded substantially. Specific practices include developing employment status prediction models to identify students at risk of employment difficulties [28], forecasting student academic performance [29] predicting university graduate employment rates through a hybrid of SVM and ant colony algorithms [30], and building graduate salary grade prediction models using deep learning algorithms [31]. In addition, algorithms such as CatBoost [32] and RF [29,33,34] have also been widely adopted in student employment prediction research, providing important methodological references for large-scale data modelling in the education domain.

Despite these research advances, data mining technologies applied to student employment prediction still present notable limitations. A systematic and complete analytical framework for employment prediction based on students’ basic attributes and training process data has not yet been established. Although extensive discussions have been conducted in academia regarding student employment issues [11,25,35], most existing studies have concentrated on theoretical dimensions such as feature engineering and model architecture design [6,8,36]. Most critically, few studies have focused on the prediction of fine-grained multi-class employment destinations, leaving a critical research gap in the precise classification of graduates’ career outcomes. To address these gaps, this study systematically explores student growth and developmental behaviour data to construct fine-grained employment destination prediction models. It further examines the intrinsic associations between the training process and employment outcomes, and on this basis, proposes targeted and feasible countermeasures to improve the employment quality of tertiary vocational college students.

## Methodology

The studies involving student’s information were approved by Hainan College of Economics and Business. The studies were conducted in accordance with the local legislation and institutional requirements. Written informed consent from the patients/participants OR patients/participants legal guardian was not required to participate in this study in accordance with the national legislation and the institutional requirements. Written informed consent was obtained from the college, for the publication of any potentially identifiable images or data included in this article.

### Research strategy

This study takes a tertiary vocational college in China as an example, applying machine learning methods to predict student employment destinations and to thoroughly investigate the associations between these destinations and various influencing factors. The study focuses on sample feature selection, the subdivision of prediction targets, and the construction of prediction models. Firstly, in response to the challenge of high-dimensional features in small datasets, RFE was adopted to select the most informative feature subset. Secondly, TPE was used for automated hyperparameter optimization. Finally, SHAP values were employed to interpret the model predictions at both global and destination-specific levels.

### Research algorithm

The XGBoost is an ensemble learning model based on the gradient boosting framework and supports parallel computation [37]. It is characterized by high training efficiency, excellent prediction accuracy, good flexibility, and strong robustness [38]. In recent years, this model has been successfully applied in various fields such as employment [25], finance [39] and energy [40], and achieved strong performance.

The XGBoost employs a greedy algorithm for node splitting: this method iterates over each feature and all its possible values, calculates the gain before and after the split, and selects the feature and split point with the maximum gain as the optimal splitting strategy for the current node.

In the XGBoost model with CART as the base learner, the computational complexity of building a single tree is O(n,d,k), where n is the number of samples, d is the number of features, and k is the maximum depth of the tree. The XGBoost explicitly introduces a regularization term to control model complexity, thereby effectively suppressing overfitting and improving generalization ability. Considering its algorithm structure and optimization strategy comprehensively, the overall computational complexity of the XGBoost lies between O(n,d,k) and O(n,d,k,logn).

### TPE hyperparameter optimization method

Hyperparameters are a set of parameters that need to be predefined before model training (such as the number of CARTs, learning rate, etc.). They are used to define the model structure and regulate the learning process, having a key impact on the performance of machine learning models. Hyperparameter optimization aims to systematically adjust these parameters to maximize the model’s predictive performance. Compared with traditional methods such as random search and grid search, automatic hyperparameter optimization can establish a mapping relationship between parameters and model performance, significantly reducing the number of tuning times and improving efficiency. This study employs a variant of Bayesian optimization TPE to achieve automatic optimization of XGBoost hyperparameters.

TPE is an optimization algorithm based on sequential models, which has good convergence ability and global exploration performance [41]. It can efficiently and finely search for the optimal hyperparameter region. The algorithm models the hyperparameter space by transforming it into a nonparametric density distribution, specifically implementing the following three types of distribution transformations [42]:

(1) Uniform distribution: Truncated Gaussian mixture distribution;
(2) Log uniform distribution: Exponential truncated Gaussian mixture distribution;
(3) Discrete distribution: Reweighted discrete distribution.

TPE uses different observations to model in the nonparametric density, which can adapt to various density based learning algorithms. Its density function is defined as follows (Eq. 1):


$$\begin{array}{c} p(x|y)=\left\{ \begin{array}{c} l(x)\;if\;y\;<\;y^{*}\\ g(x)\;if\;y\;\geq y^{*} \end{array}\right.\; \end{array}$$ (1)

Where, l(x) denotes the density function corresponding to observations with objective function values below the threshold $y^{*}$, while $y^{*}$ corresponds to observations with target values greater than or equal to $y^{*}$ Generally, TPE employs the $y^{*}$ quantile of the observed values as the threshold [42]. The relevant parameter settings for TPE Hyperparameters are shown in Table 1.

**Table 1:** TPE hyperparameter search space.

Parameter	Type	Search Range	Distribution
n_estimators	Integer	[100, 300]	Uniform
max_depth	Integer	[3, 12]	Uniform
learning_rate	Continuous	[0.01, 0.3]	Log-Uniform
subsample	Continuous	[0.7, 1.0]	Uniform
colsample_bytree	Continuous	[0.5, 1.0]	Uniform
min_child_weight	Integer	[1, 10]	Uniform
reg_alpha (L1)	Continuous	[0.0, 2.0]	Uniform
reg_lambda (L2)	Continuous	[0.1, 5.0]	Uniform
gamma	Continuous	[0.0, 0.1]	Uniform

The number of TPE iterations was set to 100 [39,41] for guidance on iteration count. To enable a fair comparison, grid search and random search were also implemented alongside the TPE tuning scheme, with identical hyperparameter search ranges, a fixed total of 100 search iterations, and a unified random seed of 42 adopted across all three optimization strategies. All three optimization approaches shared the same 5-fold stratified cross-validation setup on training subsets to rule out random split interference. Measured outcomes of the three tuning strategies were sorted into Table 2 for intuitive comparison, from which we can spot the three optimization strategies achieve comparable macro-F1 scores on the held-out test set (TPE: 0.5263, Grid Search: 0.5057, Random Search: 0.5269). This indicates that for this dataset, all three methods are capable of finding good hyperparameter configurations, with TPE providing a competitive alternative to traditional search methods.

**Table 2:** Comparison results of three optimization schemes.

Methods	Accuracy	Precision	Recall	Specificity	Macro-F1
TPE-Optimized	0.8172	0.6608	0.4880	0.7659	0.5263
Grid Search	0.8260	0.6265	0.4750	0.7695	0.5057
Random Search	0.8216	0.7244	0.4835	0.7625	0.5269

The algorithm maintains an ordered list of observations in the observation domain, ensuring that the time complexity of each iteration grows linearly with the sample size and the optimized feature dimension. The expression for expected improvement (EI) is as follows (Eq. 2):


$$\begin{array}{c} {EI}_{y^{*}}(x)=\int_{-\infty }^{y^{*}}(y^{*}-y)p(y|x)dy=\int_{-\infty }^{y^{x}}(y^{*}-y)\frac{p(x|y)p(y)}{p(x)}dy \end{array}$$ (2)

Where, $y^{*}$ denotes the threshold; setting $\gamma =p(y<y^{*})$, specifies a particular quantile of the TPE algorithm that is used to partition l(x) and g(x), with its value constrained to lie within the interval (0, 1). This ensures that each iteration returns a value $x^{*}$ that maximizes the expected improvement (Eq. 3).


$$\begin{array}{c} x^{*}=argmax\;{EI}_{y^{*}}(x) \end{array}$$ (3)

## Data source

To gain an in–depth understanding of the employment destinations of different types of tertiary vocational college students, we obtained comprehensive administrative records for all graduates between 2016 and 2019 cohorts from the Academic Affairs Office, the Student Affairs Department, and the China Volunteer Service Net about 3 weeks in January 2024. Researchers reported the purpose of data collection to the institution, and appropriate data anonymization was applied in compliance with ethical guidelines. The final cleaned dataset comprises 2,270 student records (N = 2,270), representing the complete graduate population of the institution during the study period, which minimizes selection bias. The employment destination label was determined based on official records collected 6 months post-graduation, categorized into three classes. The raw administrative records originally distinguished four destination categories: (1) Smooth Employment (78.77%, n = 1,788); (2) Job-Waiting (3.30%, n = 75); (3) Further Study (17.80%, n = 404); and (4) Non-employment (0.13%, n = 3). Because Job-Waiting and Non-employment both represent unemployment post-graduation trajectories, and the extreme sparsity of these two minority classes compromised the reliability of per-class evaluation and model estimation, the Job-Waiting category and Non-employment was merged with Unemployment. The consolidated three-class scheme used throughout this study is: (1) Smooth Employment (78.77%, n = 1,788); (2) Further Study (17.80%, n = 404); and (3) Unemployment (3.43%, n = 78).

Categorical encoding: Nominal categorical variables (e.g., gender, ethnicity, major name, region type, general difficulty student, particlularly difficultsy studnet, school skill competition, province skill competition, country skill competition, outstanding student, outstanding student leader, employment destinations) were encoded using ordinal/label encoding, which is directly compatible with tree-based models such as XGBoost. Numerical variables (e.g., Gaokao scores, college English test band4, college English test band6, first semester GPA, second semester GPA, third semester GPA, forth semester GPA, fifth semester GPA, sixth semester GPA, over GPA, volunteer service hours, amount of assistance) are normalized using a linear scaling method to map their values onto the interval [0, 1].

This study collected research data from three official campus management systems. Every selected data item is set as mandatory content in daily information registration, which means no missing values exist in the original samples. Accordingly, it is unnecessary to adopt relevant imputation strategies in subsequent analysis. All GPA features (X_18_–X_23_, representing semesters 1–6) reflect in-school academic records collected prior to graduation and prior to the employment survey. The employment destination label was recorded post-graduation, ensuring no temporal leakage. The data pipeline (imputation, scaling, RFE) was fitted exclusively on the training set (80% split) and applied to the test set without re-fitting.

To identify key factors influencing student employment, this study removed invalid features, highly redundant features, and data items with high missing rates from the original datasets. The final structure and coding of educational datasets comprises 27 features, as shown in Table 3.

**Table 3:** Variable description and encoding.

Feature Type	Type	Variable	Code	Range	Min	Max	Mean	Std. Deviation
Input Feature	Basic	Gender	$X_{\text{1}}$	1	1	2	1.81	0.39
		Age	$X_{\text{2}}$	8	19	27	22.02	1.05
		Ethnicity	$X_{\text{3}}$	1	1	2	1.28	0.45
		Major Name	$X_{\text{4}}$	6	1	7	1.90	1.27
		Gaokao scores	$X_{\text{5}}$	441.01	284.01	725.01	497.90	36.66
		Regional Type	$X_{\text{6}}$	1	1	2	1.37	0.48
		General difficulty student	$X_{\text{7}}$	1	1	2	1.29	0.46
		Particularly difficulty student	$X_{\text{8}}$	1	1	2	1.23	0.42
	Honor	School Skill Competition	$X_{\text{9}}$	1	1	2	1.00	0.051
		Province Skill Competition	$X_{\text{1}\text{0}}$	1	1	2	1.01	0.10
		Country Skill Competition	$X_{\text{1}\text{1}}$	1	1	2	1.00	0.06
		Outstanding student	$X_{\text{1}\text{2}}$	11	1	2	1.07	0.25
		Outstanding student leader	$X_{\text{1}\text{3}}$	1	1	2	1.03	0.16
		Total Scholarship	$X_{\text{1}\text{4}}$	6350	0	6350	24.01	256.48
	Certification Situations	National Computer Rank Examination	$X_{\text{1}\text{5}}$	2	0	2	0.59	0.518
		College English Test Band4	$X_{\text{1}\text{6}}$	493	0	493	93.03	147.61
		College English Test Band6	$X_{\text{1}\text{7}}$	439	0	439	0.86	17.00
	GPA	First Semester GPA	$X_{\text{1}\text{8}}$	2270	2.81	1.34	4.15	2.934
		Second Semester GPA	$X_{\text{1}\text{9}}$	2270	3.51	0.73	4.24	2.808
		Third Semester GPA	$X_{\text{2}\text{0}}$	2270	3.43	0.85	4.28	3.019
		Forth Semester GPA	$X_{\text{2}\text{1}}$	2270	4.4	0.24	4.64	3.087
		Fifth Semester GPA	$X_{\text{2}\text{2}}$	2270	4.49	0	4.49	3.021
		Sixth Semester GPA	$X_{\text{2}\text{3}}$	2270	4.88	0	4.88	3.184
		Over GPA	$X_{\text{2}\text{4}}$	2.99	1.22	4.21	2.99	0.35
	Others	Volunteer Service Hours	$X_{\text{2}\text{5}}$	660.40	0.00	660.40	75.82	63.54
		Amount of Assistance	$X_{\text{2}\text{6}}$	11400.00	0.00	11400.00	2686.92	3687.55
Output Feature	Employment	Employment Destinations	$X_{\text{2}\text{7}}$	3	0	3	1.32	0.598

## Results and discussion

### Feature selection and hyperparameter analysis

In machine learning tasks, highly correlated independent variables can easily lead to overfitting. However, such issues are typically manageable by the algorithms themselves, and correlation analysis is relatively straightforward in this context. Nevertheless, noise variables that are irrelevant to the prediction target not only increase model complexity and training burden but also reduce model interpretability.

This study divided the preprocessed data into training and testing sets at a ratio of 8:2 (training: n = 1816, test: n = 454). Given that the educational datasets used in this study is large scale, structurally complex, and contains both discrete and continuous features with considerable redundancy, the curse of dimensionality is likely to occur in high dimensional scenarios. Therefore, feature selection is particularly crucial. This study employed RFE with XGBoost as the base estimator.

RFE was implemented using scikit-learn’s RFE class with XGBoost as the base estimator. At each iteration, the least important feature (based on XGBoost’s feature importance scores) was eliminated. The optimal feature count was determined via stratified 5-fold cross-validation on the training set, selecting the subset that maximized macro-averaged F1 score. To assess feature stability, RFE was repeated across 50 bootstrap samples of the training set; features appearing in the optimal subset in ≥80% of bootstrap iterations were considered stable. All 22 features selected in the final model met this stability criterion (shown in Table 4). Importantly, RFE was performed exclusively within the training fold of each cross-validation split to prevent data leakage.

**Table 4:** Selected features for optimal model.

Rank	Selected Features	Rank	Selected Features
1	$X_{\text{4}}$	12	$X_{\text{2}\text{2}}$
2	$X_{\text{1}\text{6}}$	13	$X_{\text{2}\text{1}}$
3	$X_{\text{5}}$	14	$X_{\text{2}\text{4}}$
4	$X_{\text{1}}$	15	$X_{\text{2}\text{3}}$
5	$X_{\text{2}}$	16	$X_{\text{1}\text{5}}$
6	$X_{\text{2}\text{5}}$	17	$X_{\text{1}\text{2}}$
7	$X_{\text{1}\text{9}}$	18	$X_{\text{2}\text{6}}$
8	$X_{\text{7}}$	19	$X_{\text{3}}$
9	$X_{\text{6}}$	20	$X_{\text{8}}$
10	$X_{\text{1}\text{8}}$	21	$X_{\text{1}\text{3}}$
11	$X_{\text{2}\text{0}}$	22	$X_{\text{1}\text{4}}$

Fig 1 illustrates how model performance varies with the number of features during the RFE process. As the number of features increases from 1 to 27, the macro-averaged F1-score generally improves, reflecting the contribution of informative predictors. The performance reaches a strong local optimum at 22 features (macro-F1 = 0.5228), after which adding further features yields diminishing or fluctuating returns and eventually a decline, indicating that noise interference intensifies beyond this point. Based on this, the optimal number of features was determined to be 22. Feature stability was verified using the 50-bootstrap procedure described above: all 22 selected features met the ≥ 80% selection-frequency criterion across the bootstrap samples, with a stable ranking, confirming the reliability of the feature selection.

**Fig 1:**
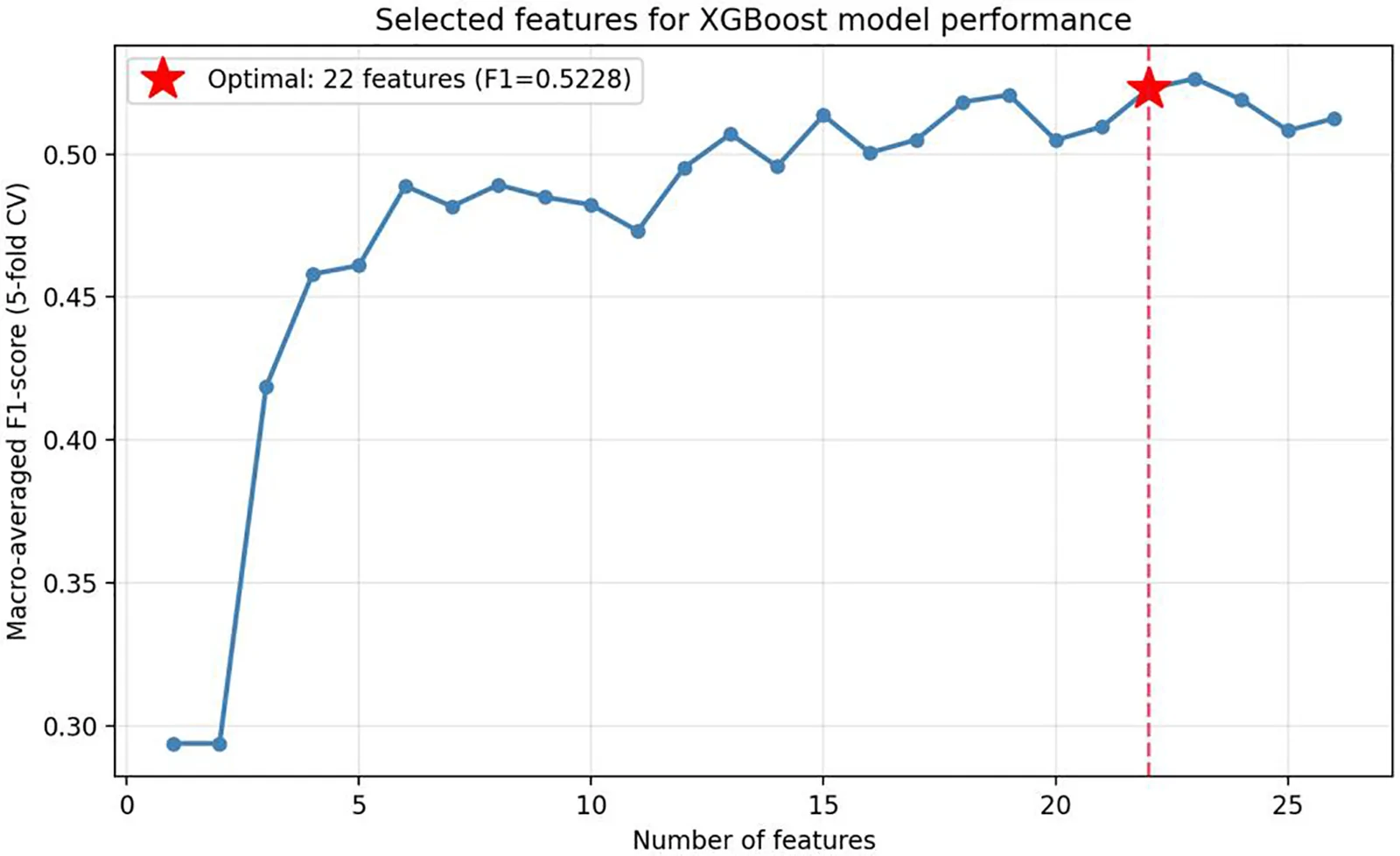
Selected features for XGBoost model performance.

The ranking of each feature variable is shown in Table 4.

On the basis of determining the optimal feature set, the TPE method was further employed for hyperparameter optimization to enhance model performance. During model evaluation, a confusion matrix was constructed based on the true classes of samples and prediction results. To comprehensively assess the discrimination performance of the classification model both overall and for each category, this study utilized five complementary evaluation metrics: Accuracy, Precision, Recall (Sensitivity), Specificity, and F1-Score.The selection of these metrics is justified by the multi-class nature of the prediction task and the presence of class imbalance: Accuracy provides an overall view; Precision and Recall capture class-specific performance; Specificity measures the true-negative rate; and macro-averaged F1 balances precision and recall across all classes regardless of class frequency (Eqs. 4–8):


$$\begin{array}{c} Accuracy=\frac{TP+TN}{TP+TN+FP+FN} \end{array}$$ (4)

$$\begin{array}{c} Precision=\frac{TP}{TP+FP} \end{array}$$ (5)

$$\begin{array}{c} Sensitivity=Recall=\frac{TP}{TP+FN} \end{array}$$ (6)

$$\begin{array}{c} Specificity=\frac{TN}{TN+FP} \end{array}$$ (7)

$$\begin{array}{c} F\text{1}-Score=\text{2}\times \frac{Precision\times Recall}{Precision+Recall} \end{array}$$ (8)

Because this is a three-class classification problem, the binary TP/FP/TN/FN definitions were extended to the multi-class setting using a one-vs-rest (OvR) strategy: for each class c, TP_c counts samples of class c correctly predicted as c; FP_c counts samples of other classes incorrectly predicted as c; FN_c counts samples of class c predicted as another class; and TN_c counts all remaining samples. This yields a 3 × 3 confusion matrix (Shown in Table 5), and all reported precision, recall, specificity, and F1 values are macro-averaged (Shown in Table 6) across the three classes.

**Table 5:** Confusion matrix of the TPE-Optimized XGBoost model on the held-out test set (n = 454).

Actual value	Predicted value		
	Unemployment	Smooth Employment	Further Study
Unemployment	2	14	0
Smooth Employment	2	337	18
Further Study	0	49	32

**Table 6:** Class-wise performance metrics (macro-averaged, one-vs-rest) of the TPE-Optimized XGBoost model on the held-out test set.

Class	Precision	Recall	Specificity	F1-Score	Support (n)
Unemployment	0.5000	0.1250	0.9954	0.2000	16
Smooth Employment	0.8425	0.9440	0.3505	0.8904	357
Further Study	0.6400	0.3951	0.9517	0.4885	81
Macro-average	0.6608	0.4880	0.7659	0.5263	454

### Results of TPE hyperparameter optimization

#### TPE-Optimization trajectory and objective score distribution.

Fig 2(A) illustrates the optimization trajectory of the TPE algorithm across 100 trials, where the horizontal axis represents the trial index and the vertical axis denotes the macro-averaged F1-score (F1_macro), the optimization objective metric for the XGBoost classifier. The model performance fluctuates continuously throughout the searching process, with objective values ranging approximately from 0.46 to 0.52. The optimal trial, highlighted with a red start at trial 22, achieves the maximum objective value of 0.5228, demonstrating that the TPE sampler effectively explores the hyperparameter space and successfully locates a superior hyperparameter combination. Fig 2(B) presents the histogram distribution of all objective scores generated in the 100 search trials. The objective values are primarily distributed within the range of 0.48 to 0.52, with the majority of trials clustering between 0.49 and 0.52. The vertical dashed red line marks the best objective value (0.5228) obtained in the optimization process. The distribution reveals that only a small proportion of hyperparameter configurations yield competitive performance close to the optimal score, which validates the necessity of systematic hyperparameter searching instead of arbitrary manual parameter tuning.

**Fig 2:**
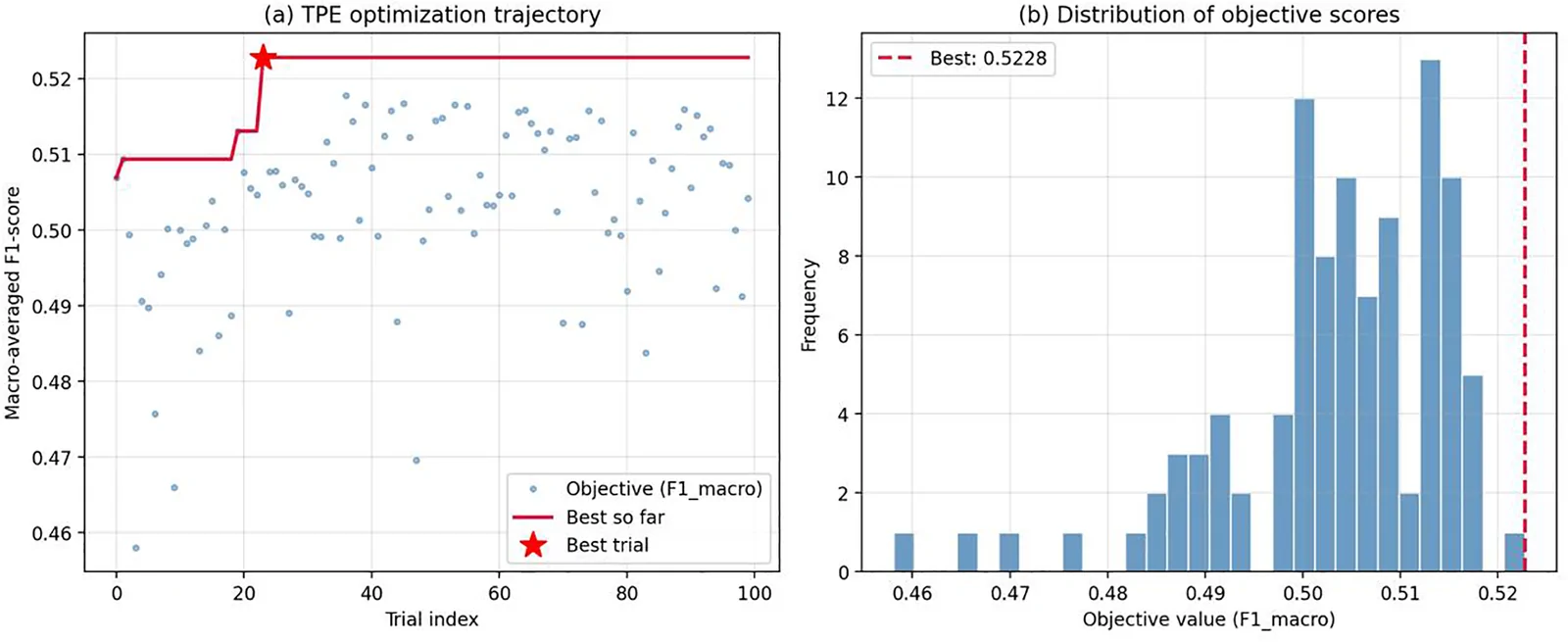
TPE optimization analysis for the XGBoost model: (a) optimization trajectory of the objective (F1_macro) across 100 trials, with the best trial highlighted; (b) distribution of objective scores over all trials.

#### Relationships between individual hyperparameters and model performance.

To provide a robust estimate of model performance and quantify uncertainty, stratified 5-fold cross-validation was conducted on the full training set. The number of iterations for hyperparameter optimization was increased to 100, following recommendations from [39,41], ensuring sufficient convergence of the TPE optimizer.

As illustrated in Fig 3, the scatter plot matrix visualizes the pairwise relationships between each XGBoost hyperparameter and the F1_macro objective metric. Each blue dot corresponds to one trial configuration sampled during TPE optimization, and the red star denotes the optimal hyperparameter set identified in the best trial. Clear patterns can be observed for critical hyperparameters. For n_estimators, higher values generally correspond to improved model performance, and the optimal configuration lies within the range of 225–275. The best learning_rate is found around 0.14; overly low or high learning rates degrade prediction performance. The optimal subsample value approaches 0.8, indicating that sampling a large fraction of training samples facilitates stable model training. Meanwhile, hyperparameters including max_depth, min_child_weight, reg_alpha, reg_lambda, colsample_bytree, and gamma exhibit non-monotonic relationships with the objective score. This non-linear characteristic confirms that grid search and random search are inefficient for this modelling task, further justifying the adoption of Bayesian TPE optimization.

**Fig 3:**
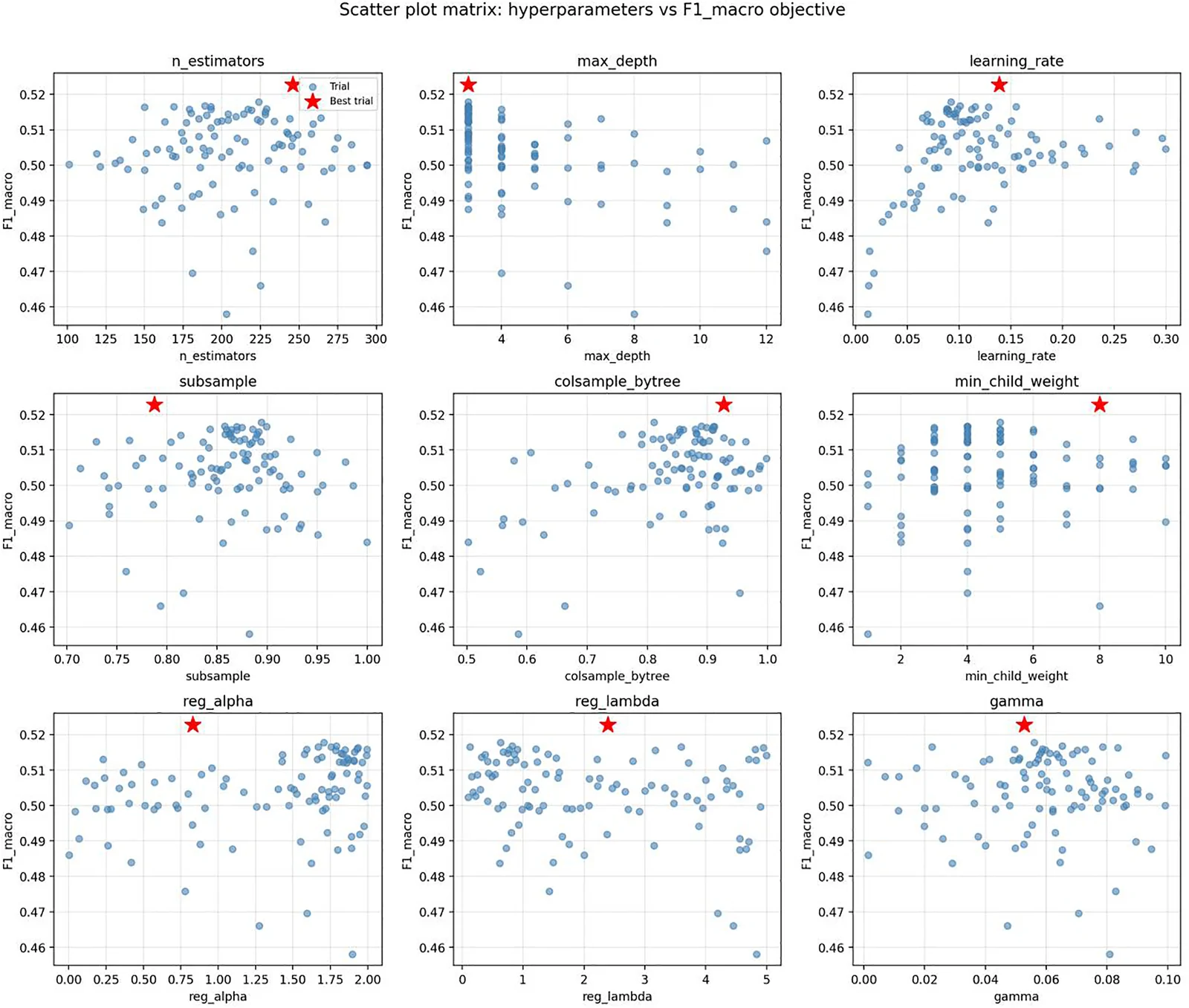
The process using the XGBoost model for TPE hyperparameter optimization.

As illustrated in Fig 3, during the hyperparameter optimization process, the model achieved a maximum F1_macro value of 0.5228, indicating its good predictive performance and demonstrating that the model constructed in this study can be effectively applied to the prediction of tertiary vocational college students’ employment destinations. The hyperparameter configuration corresponding to this optimal F1 value is shown in Table 7.

**Table 7:** TPE hyperparameter configuration of the XGBoost model.

No	TPE Hyperparameter Name	TPE Hyperparameter Value	Describe
1	colsample_bytree	0.92663243	Random sampling fraction of features per tree
2	gamma	0.05266919	Minimum loss reduction required to split a node (complexity penalty term)
3	learning_rate	0.13851021	Step size shrinkage factor for each boosting iteration
4	max_depth	3	The maximum depth of single decision tree, controls tree complexity
5	min_child_weight	8	Minimum sum of instance weights (hessian) in a leaf node
6	n_estimators	246	Total number of decision trees in the ensemble
7	reg_alpha	0.82918351	L1 regularization term on leaf weights
8	reg_lambda	2.39267171	L2 regularization term on leaf weights
9	subsample	0.78736723	Random sampling fraction of training samples per tree

#### Spearman correlation between hyperparameters and objective metric.

The Spearman rank correlation heatmap quantifies the monotonic correlations among all hyperparameters and the F1_macro objective(as shown in Fig 4). The correlation coefficient ranges from −1 to +1, where red cells represent positive correlations and blue cells represent negative correlations. In terms of correlations with the target objective F1_macro:

**Fig 4:**
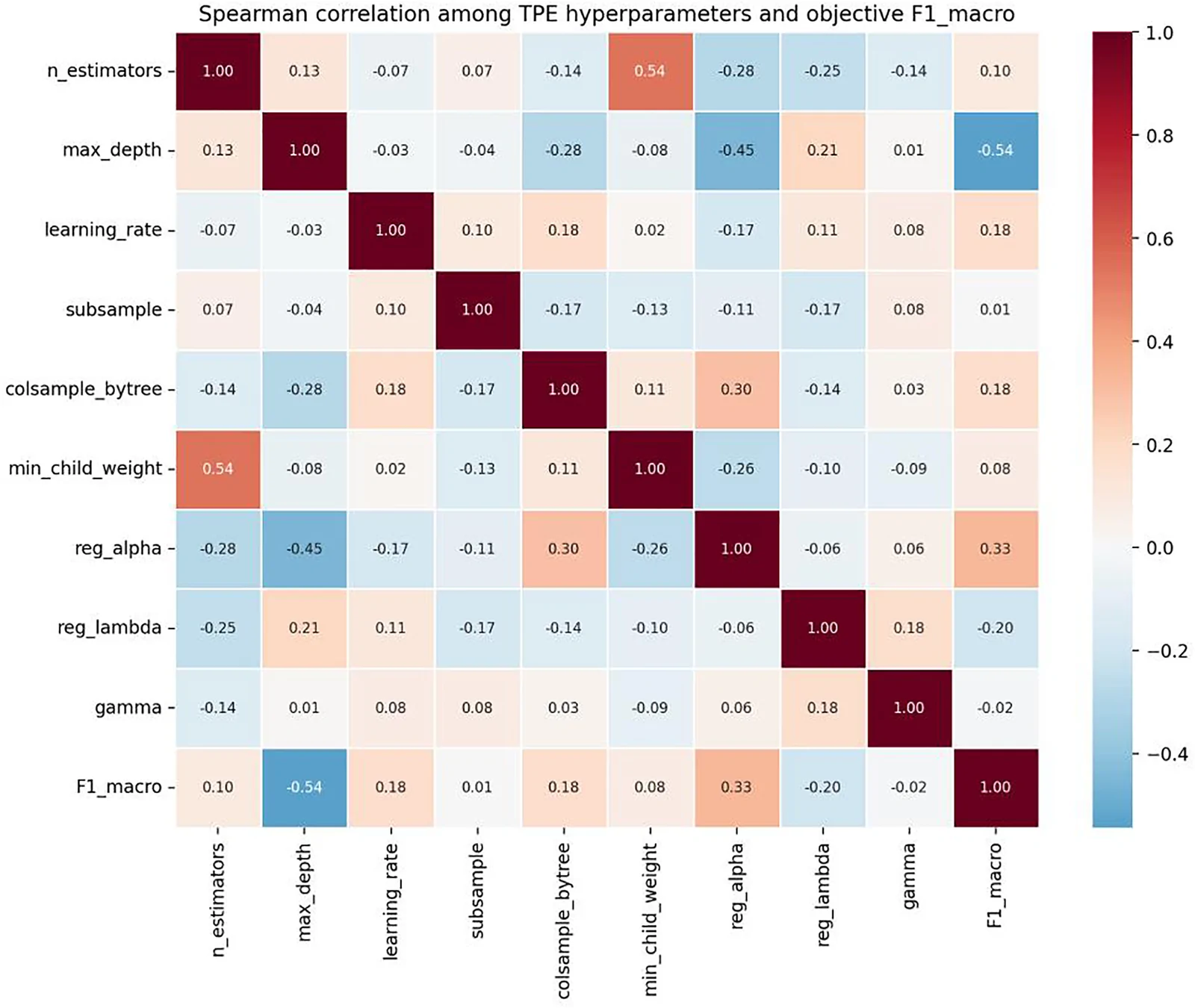
Spearman correlation among TPE hyperparameters and the objective F1_macro.

max_depth shows a strong negative correlation (r = −0.54), suggesting that excessively deep decision trees impair generalization performance;

reg_alpha (r = 0.33) shows a moderate positive correlation, while learning_rate (r = 0.18), colsample_bytree (r = 0.18), and n_estimators (r = 0.10) exhibit weak positive correlations with model performance;

Other regularization hyperparameters (gamma, reg_lambda, min_child_weight, subsample) show weak correlations with the final prediction performance. Significant cross-correlations also exist between certain hyperparameters. These mutual dependencies imply that hyperparameters cannot be tuned independently, which reinforces the advantage of the TPE Bayesian optimization framework that jointly optimizes the full hyperparameter space.

### Model comparison

Before contrasting different algorithms, all comparative models such as LR, DT, SVM, KNN, LightGBM and RF got their own hyperparameters tuned via random search plus stratified 5-fold cross validation under consistent experimental rules. Such unified tuning settings helped eliminate unfair comparison stemming from inconsistent parameter optimization depth, and relevant notes were added under Table 8.

**Table 8:** Comparison of proposed model with other models.

Model	Accuracy	Precision	Recall	Specificity	F1
TPE-Optimized XGBoost	0.8172	0.6608	0.4880	0.7659	0.5263
Non-Optimized XGBoost	0.8194	0.5120	0.4428	0.7593	0.4593
Random Forest	0.8282	0.5684	0.4211	0.7396	0.4386
LightGBM	0.8172	0.6616	0.4649	0.7607	0.4942
Decision Tree	0.8106	0.4942	0.4327	0.7480	0.4464
Logistic Regression	0.8106	0.8128	0.4335	0.7401	0.4617
K-Nearest Neighbor	0.8062	0.5902	0.4547	0.7415	0.4858
Support Vector Machine	0.7599	0.4684	0.4629	0.7689	0.4633

Table 8 shows that the TPE-Optimized XGBoost consistently outperforms all baseline models across most key metrics on the held-out test set (n = 454). It achieves the highest macro-averaged F1-score (0.5263), macro-averaged Recall (0.4880), and balanced accuracy (0.4880), with a competitive macro-averaged Precision (0.6608). In contrast, the non-optimized XGBoost and other traditional models (RF, LightGBM, Decision Tree, Logistic Regression, KNN, SVM) exhibit lower overall performance, especially in Recall and F1-score, which are critical for imbalanced multi-class prediction. These findings confirm that TPE hyperparameter optimization significantly enhances XGBoost’s predictive capacity and generalization for fine-grained employment destination prediction.

### Employment destinations analysis

#### Smooth employment.

Analysis of the research data reveals that a significant proportion of students graduating from junior colleges can secure direct employment, accounting for 78.8% of the datasets. Fig 5 displays the key features affecting students’ smooth employment status and their contribution to the prediction model output. In terms of feature importance ranking, major name (X_4_) has the highest predictive power, followed by Gaokao scores (X_5_), forth semester GPA (X_21_), volunteer service hours (X_25_), and gender (X_1_), indicating that major orientation, initial academic foundation, mid-program academic performance and social practice experience play a core role in determining whether students achieve smooth employment.

**Fig 5:**
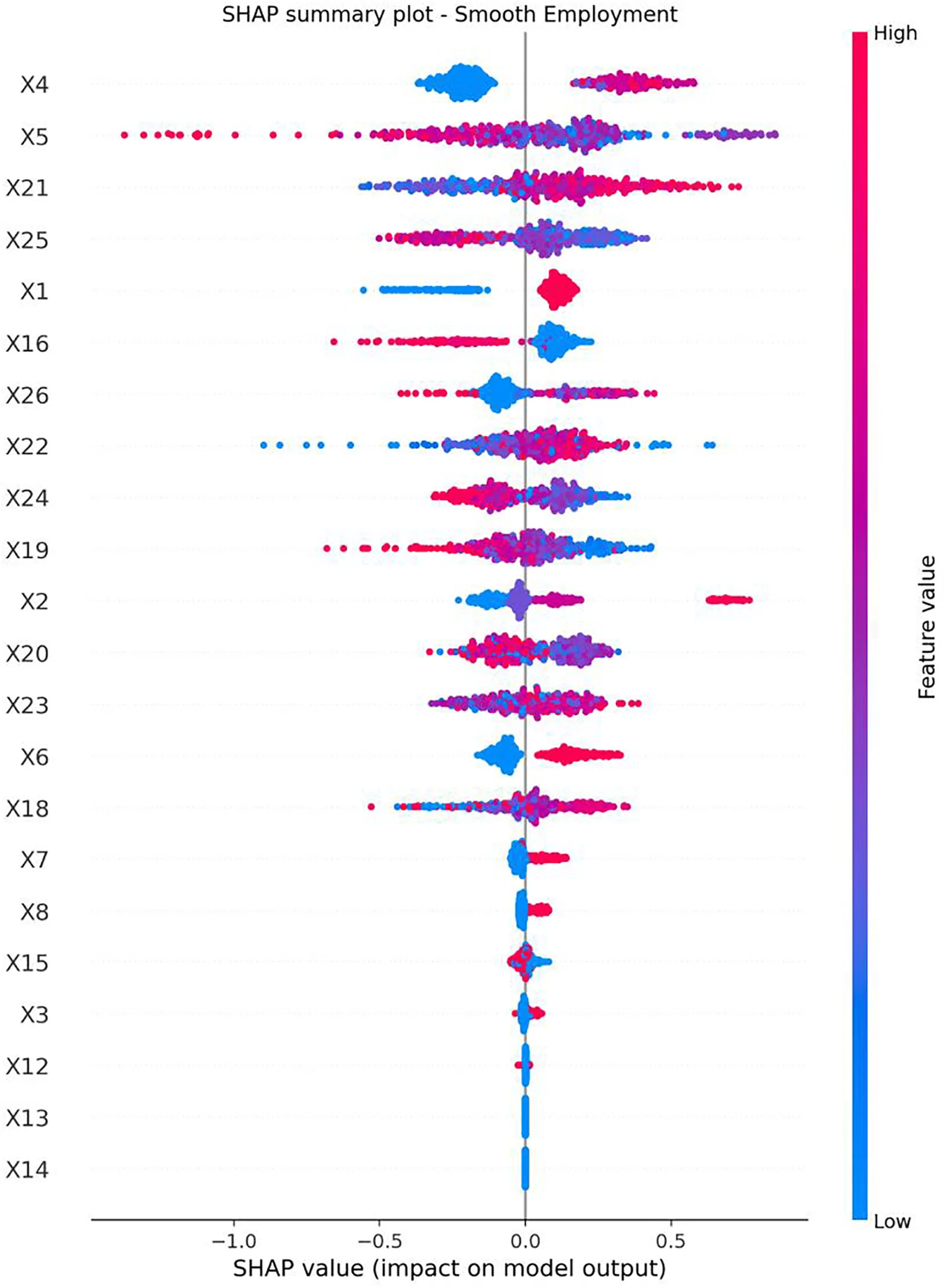
SHAP beeswarm plots of smooth employment students.

Specifically, a positive SHAP value indicates that the feature will increase the probability of achieving smooth employment, while a negative value indicates the opposite. Combined with the feature value color mapping (red for high values, blue for low values), it can be further seen that different major name categories (X_4_, with both red and blue distributions) are associated with divergent SHAP values, showing that major matching with labor market demand strongly shapes the likelihood of smooth employment. Similarly, higher Gaokao scores (X_5_) and longer volunteer service hours (X_25_) generally correspond to positive SHAP values, further confirming the positive role of solid foundational academic ability and social practice in securing smooth employment.

Although college English test band4 (X_16_) and amount of assistance (X_26_) still have some influence, their ranking has significantly dropped compared with the further study group, indicating that for smooth employment status, major category and comprehensive academic and practical indicators (such as X_4_, X_5_, X_21_, X_25_) are more critical than English proficiency. Factors such as outstanding student leader (X_13_), total scholarship (X_14_) are at the bottom of the list, indicating that their contribution to the judgment of smooth employment status is relatively limited.

In addition, the impact of regional type (X6) is also quite evident, with significant differences in the probability of smooth employment across different regional backgrounds, reflecting the importance of regional labor market characteristics on graduate employment outcomes.

#### Unemployment.

Fig 6 illustrates the key features affecting students’ unemployment status and their contribution to the prediction model output. In terms of feature importance ranking, fifth semester GPA (X_22_) has the highest predictive power, followed by third semester GPA (X_20_), sixth semester GPA (X_23_), volunteer service hours (X_25_), and second semester GPA (X_19_), indicating that mid-to-late academic performance and social practice experience play a core role in determining whether students face unemployment.

**Fig 6:**
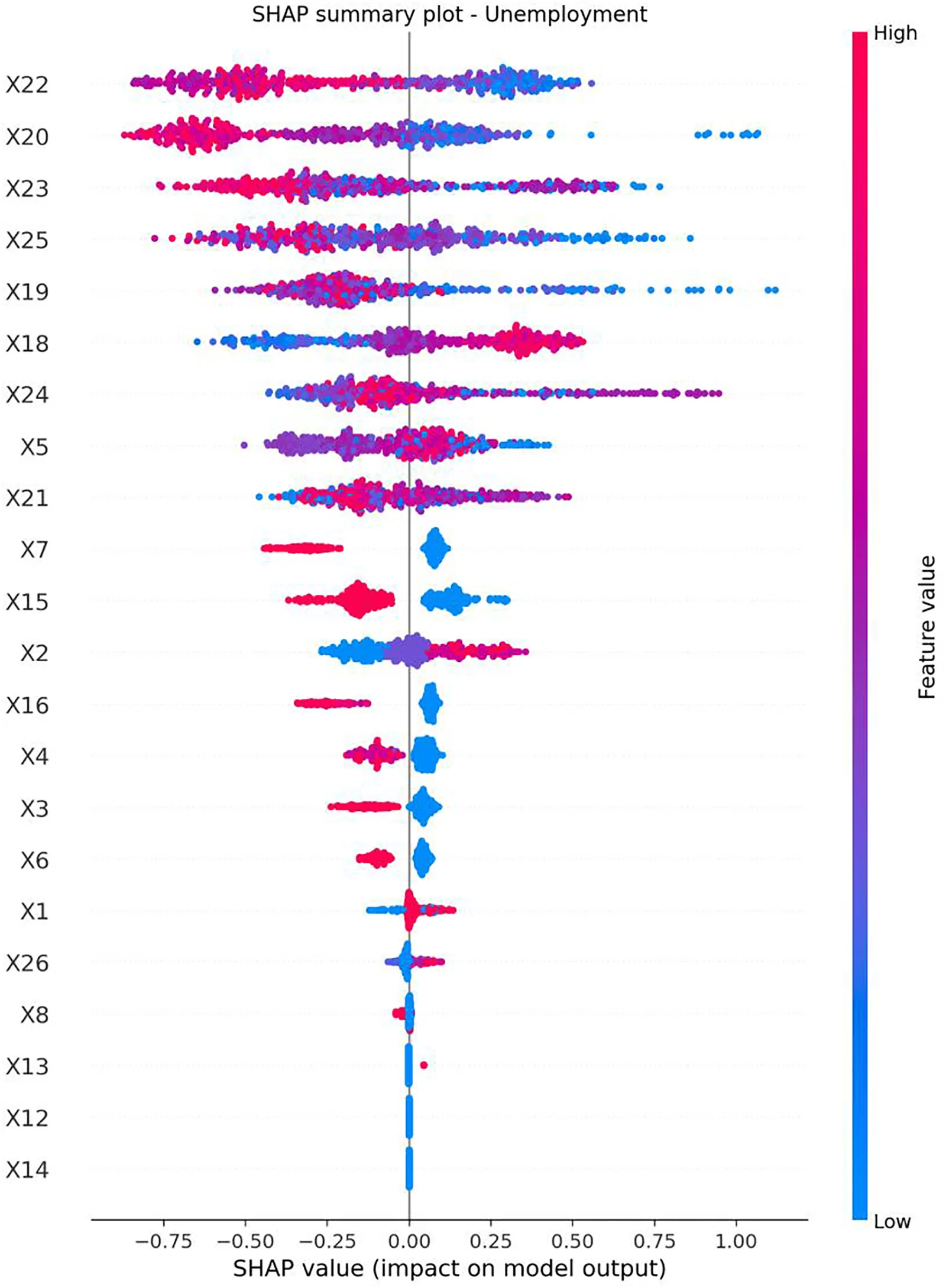
SHAP beeswarm plots of unemployment students.

Specifically, a positive SHAP value indicates that the feature will increase the probability of being unemployed, while a negative value indicates the opposite. Combined with the feature value color mapping, it can be further seen that lowerfifth semester GPA (X_22_, mostly shown in blue) is significantly associated with positive SHAP values, indicating that poor mid-to-late academic performance can effectively raise the risk of unemployment. Similarly, shorter volunteer service hours (X_25_) generally correspond to positive SHAP values, further confirming the protective role of social practice in avoiding unemployment.

On the other hand, although Gaokao scores (X_5_) and forth semester GPA (X_21_) still have some influence, their ranking has significantly dropped compared with the smooth employment group, indicating that for unemployment status, mid-to-late semester academic performance (such as X_22_, X_20_, X_23_) is more critical than initial academic foundation. Factors such as outstanding student leader (X_13_), outstanding student (X_12_) and total scholarship (X_14_) are at the bottom of the list, indicating that their contribution to the judgment of unemployment status is relatively limited.

The impact of general difficulty student (X_7_) is also quite evident, with significant differences in the probability of unemployment across different student hardship groups, reflecting the importance of student support status on graduate employment outcomes.

#### Further study.

Fig 7 illustrates the key features affecting student’ further study status and their contribution to the prediction model output. In terms of feature importance ranking, college English test band4 (X_16_) has the highest predictive power, followed by Volunteer Service Hours (X_25_), Gaokao scores (X_5_), first semester GPA (X_18_), and second semester GPA (X_19_), indicating that English proficiency, initial academic foundation, early-stage academic performance and social practice experience play a core role in determining whether students choose further study.

**Fig 7:**
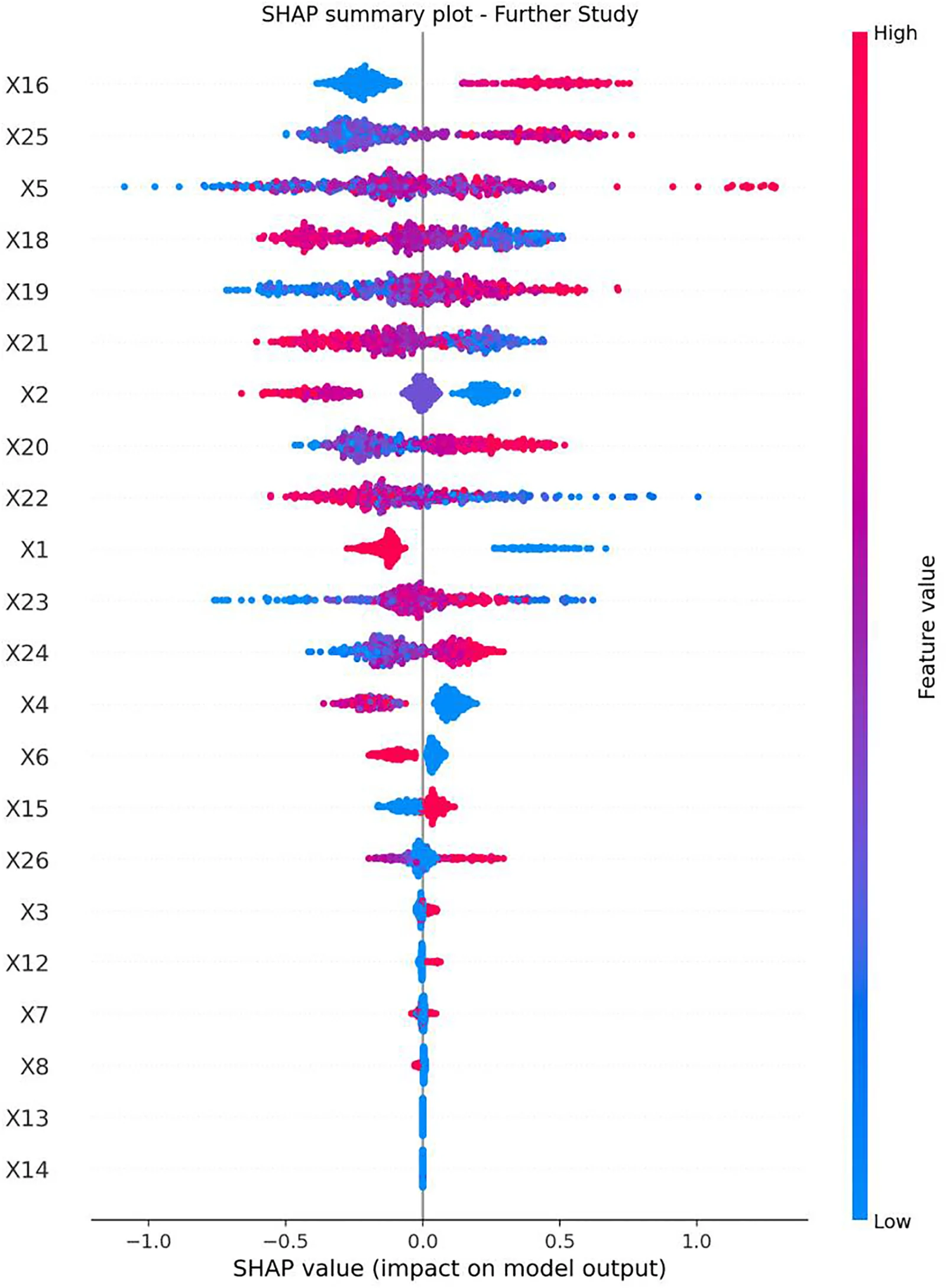
SHAP beeswarm plots of further study students.

Specifically, a positive SHAP value indicates that the feature will increase the probability of choosing further study, while a negative value indicates the opposite. Combined with the feature value color mapping, it can be further seen that higher college English test band4 scores (X_16_, mostly shown in red) are significantly associated with positive SHAP values, indicating that better English proficiency can effectively raise the likelihood of pursuing further study. Similarly, longer volunteer service hours (X_25_) generally correspond to positive SHAP values, further confirming the positive role of social practice in students’ choice of further education.

Although fifth semester GPA (X_22_) and sixth semester GPA (X_23_) still have some influence, their ranking has significantly dropped compared with the unemployment group, indicating that for further study status, early academic indicators (such as X_16_, X_5_, X_18_, X_19_) are more critical than mid-to-late semester academic performance. Factors such as outstanding student leader (X_13_), total scholarship (X_14_) are at the bottom of the list, indicating that their contribution to the judgment of further study status is relatively limited.

The impact of gender (X_1_) is also quite evident, with significant differences in the probability of pursuing further study across different gender groups, reflecting the importance of individual demographic characteristics on students’ decisions of continuing education.

## Conclusion, implication and limitation

### Conclusion

This study employs seven distinct machine learning methods to predict the employment destinations of students from a tertiary vocational college in China, and also explores the potential of identifying key indicators in small datasets, which are used to construct predictive models through feature selection and hyperparameter optimization algorithms. The achievements of this study verify the effectiveness of the proposed TPE-Optimized XGBoost framework for fine-grained three classes employment destination prediction.

Firstly, compared with other main models currently in experiment, the TPE-Optimized XGBoost model utilized in this study achieves competitive predictive performance [39,43]. The TPE optimization demonstrates its effectiveness over default XGBoost is confirmed by the experiment (Shown in Table 8).

Secondly, the SHAP interpretation method was employed to elucidate the prediction results for students’ various employment destinations. All SHAP interpretations are framed as associations rather than causal claims, in accordance with the correlational nature of the study design [39].

Thirdly, the group of students who secure smooth employment is primarily positively associated with a combination of Gaokao scores, volunteer service hours, and mid-to-late-term GPA. This conclusion differs from the findings of [44], possibly because their study focused on undergraduate students, whereas this study targets tertiary vocational college students whose employment pathways are more directly influenced by practical performance.

Finally, empirical analysis demonstrates that the constructed predictive model has superior performance, and the SHAP method is used to analyze the factors associated with the three employment destinations, alleviating the difficulties of applying machine learning methods in practice.

### Implication

The unemployment status is significantly associated with mid-term academic performance. Students pursuing further studies rely more on early academic foundations (such as entrance exam scores), CET4 scores, and volunteer service experience. In contrast, those choosing unemployment typically exhibit consistently high GPA across multiple semesters, indicating proactive planning rather than passive job avoidance.

Practical Intervention Recommendations for Tertiary Vocational Colleges:

Early Warning System: Colleges should implement a real-time dashboard that flags students with GPA trajectories associated with unemployment risk (e.g., declining GPA in semesters 4–5). Personalized career counseling should be triggered automatically for at-risk students by the end of semester 3.

Volunteer Service Promotion: Given the strong positive association between volunteer service hours and both smooth employment and further study outcomes, colleges should actively encourage and facilitate volunteering programs, particularly for first-year and second-year students.

Major-Specific Career Guidance: Since major category (X_4_) significantly differentiates employment outcomes, department-level career guidance tailored to each discipline’s labor market situation should be strengthened.

Support for High-Achieving Students: Students with consistently high GPA who are associated with deferred employment may benefit from structured support for civil service exam preparation or postgraduate application, rather than generic job-seeking assistance.

Predictive Model Deployment: The TPE-XGBoost model can be deployed as a web-based tool accessible to academic advisors, who can input a student’s current profile and receive a probability distribution over the three employment destinations, enabling timely, data-driven intervention.

### Limitation

#### Sample representativeness.

The study draws on data from a single tertiary vocational college in China, covering only 2,270 graduates from the 2016–2019 cohorts. Consequently, findings derived from this institution–specific sample cannot be unconditionally generalized to the national vocational–education sector, nor do they benefit from cross–regional or cross institutional comparisons.

#### Limited variable scope.

Although the data set includes basic demographics, entrance scores, academic records, skills competition achievements and limited extracurricular engagement, it omits potentially influential factors such as family background, parental education, learning motivation, mental health, teaching quality and local labour–market conditions–variables that may exert non–trivial effects on graduate employment destinations.

#### Restricted model applicability.

The TPE-Optimized XGBoost model has been fine–tuned for granular prediction of employment outcomes within the vocational context; however, its utility for undergraduate or other educational strata remains untested. Moreover, the model is trained on historical data and lacks mechanisms for dynamically adapting to shifts in external labour–market environments. More over, this study have not tested diverse resampling methods like SMOTE to ease class imbalance of original dataset, which is left for follow-up exploration.

#### Depth of interpretability.

While the SHAP values are used to rank feature contributions, the analysis remains largely correlational. Causal pathways–such as whether volunteering hours directly enhance employability or operate indirectly through heightened soft–skill acquisition–have not been disentangled, thereby diluting the prescriptive power of the results for educational managers.

#### Representation of special populations.

The current dataset may under-represent students from disadvantaged backgrounds (e.g., students with disabilities, students from remote rural areas, or students facing financial hardship). The model’s predictions for these subgroups may be less reliable due to insufficient training examples. Future work should collect targeted data for these populations and evaluate model fairness metrics (e.g., equalized odds) to ensure equitable applicability.

As whole dataset was collected merely from a single tertiary vocational college across four graduate cohorts, this study failed to source independent out-of-sample data from other schools to carry out external validation tests at this stage. Frankly, restricted by local education data confidentiality policies and inter-college data sharing barriers, The researchers could not access graduate records from different regions or distinct types of tertiary vocational colleges. Without such outside data, this study unable to check how reliably our proposed TPE-Optimized XGBoost model could adapt to unseen student groups from separate educational environments, which inevitably constrains the generalisation credibility of our prediction outcomes. The researchers plan to tackle this gap in follow-up work by cooperating with more peer colleges to gather multi-source data and finish external validation experiments.

### Future research directions

#### Expand sample coverage and conduct multi–level validation.

Data will be collected from multiple, regionally representative tertiary vocational colleges across China, embracing diverse programme types and the newly established vocational–bachelor schools, to bolster external validity. The generalization ability of the model to the same region, different regions and different periods will be tested in the future. Parallel analyses using undergraduate employment data will be undertaken to delineate how predictors vary across educational levels.

#### Enrich the variable set and enhance model comprehensiveness.

Additional covariates– including parental education, family income, student engagement, and regional economic indicators–will be incorporated. Psychological constructs such as career–planning clarity and job–search self–efficacy will be gathered via surveys and interviews, offering a fuller account of employment–destination formation.

#### Refine model architecture and strengthen dynamic adaptability.

The TPE-Optimized XGBoost framework will be integrated with time–series techniques to create a forecasting system capable of adjusting to evolving labour–market signals. Transfer–learning protocols will be developed to recycle pretrained models across institutions, curtailing retraining costs. Hybridization with deep learning architectures (e.g., temporal convolution networks or attention–based models) will also be explored to further uplift predictive accuracy and generalisability.

## Supporting information

S1 File Dataset. (XLSX)
